# Missing steps in a staircase: a qualitative study of the perspectives of key stakeholders on the use of adaptive designs in confirmatory trials

**DOI:** 10.1186/s13063-015-0958-9

**Published:** 2015-09-28

**Authors:** Munyaradzi Dimairo, Jonathan Boote, Steven A. Julious, Jonathan P. Nicholl, Susan Todd

**Affiliations:** School of Health and Related Research, Regent Court, University of Sheffield, 30 Regent Street, S1 4DA Sheffield, UK; Centre for Research in Primary and Community Care, University of Hertfordshire, Hatfield, AL109AB, Hertfordshire, UK; Department of Mathematics and Statistics, University of Reading, Whiteknights Reading, RG6 6AX UK

**Keywords:** Adaptive designs, Flexible designs, Qualitative interviews, Confirmatory trials, Phase 3, Clinical trials, Publicly funded trials, Early stopping, Interim analyses

## Abstract

**Background:**

Despite the promising benefits of adaptive designs (ADs), their routine use, especially in confirmatory trials, is lagging behind the prominence given to them in the statistical literature. Much of the previous research to understand barriers and potential facilitators to the use of ADs has been driven from a pharmaceutical drug development perspective, with little focus on trials in the public sector. In this paper, we explore key stakeholders’ experiences, perceptions and views on barriers and facilitators to the use of ADs in publicly funded confirmatory trials.

**Methods:**

Semi-structured, in-depth interviews of key stakeholders in clinical trials research (CTU directors, funding board and panel members, statisticians, regulators, chief investigators, data monitoring committee members and health economists) were conducted through telephone or face-to-face sessions, predominantly in the UK. We purposively selected participants sequentially to optimise maximum variation in views and experiences. We employed the framework approach to analyse the qualitative data.

**Results:**

We interviewed 27 participants. We found some of the perceived barriers to be: lack of knowledge and experience coupled with paucity of case studies, lack of applied training, degree of reluctance to use ADs, lack of bridge funding and time to support design work, lack of statistical expertise, some anxiety about the impact of early trial stopping on researchers’ employment contracts, lack of understanding of acceptable scope of ADs and when ADs are appropriate, and statistical and practical complexities. Reluctance to use ADs seemed to be influenced by: therapeutic area, unfamiliarity, concerns about their robustness in decision-making and acceptability of findings to change practice, perceived complexities and proposed type of AD, among others.

**Conclusions:**

There are still considerable multifaceted, individual and organisational obstacles to be addressed to improve uptake, and successful implementation of ADs when appropriate. Nevertheless, inferred positive change in attitudes and receptiveness towards the appropriate use of ADs by public funders are supportive and are a stepping stone for the future utilisation of ADs by researchers.

**Electronic supplementary material:**

The online version of this article (doi:10.1186/s13063-015-0958-9) contains supplementary material, which is available to authorized users.

## Background

Traditionally, standard randomised controlled trials (RCTs) are designed with a fixed target sample size and recruit until this target is met. Recently, much attention has been paid to alternative types of RCTs, known as adaptive designs (ADs), in which prospectively planned modifications to the design are made based on accruing outcome data from an ongoing trial while preserving the scientific validity and integrity of that trial [1, 2]. This may mitigate the risk of making inaccurate design assumptions or potentially may shorten trial duration by allowing early stopping as soon as there is sufficient evidence to answer the research question(s) [2]. However, despite potential promising benefits to clinical trials, patients and funders, the use of ADs in practice, particularly in the public sector, has been described by advocates as disappointing - with their uptake lagging far behind methodological developments [3]. Moreover, the use of ADs is viewed as controversial, with the perception among some stakeholders that public funders and regulators have hindered their wider adoption [4].

Citing disappointing uptake, the pharmaceutical industry initiated a Pharmaceutical Research and Manufacturers of America (*PhRMA)* Adaptive Design Working Group with a vision to facilitate dialogue among key stakeholders in drug development and to establish a consensus position on the use of ADs [5]. The group further investigated barriers and opportunities associated with the use of ADs across different trial phases, specifically in drug development. Although much related discussion and research has subsequently been undertaken [2, 5–10], it has been led and driven by the pharmaceutical industry, especially in the USA, with the public sector lagging behind.

Researchers have highlighted that the public sector has its own unique multifaceted challenges, which need to be explored in detail and addressed in order to improve uptake of ADs [2, 11, 12]. With this in mind, the NIH (National Institutes of Health, USA) and associates funded and facilitated a 2-day workshop to initiate some cross-industry discussions with representatives from the NIH, the Food and Drug Administration (FDA), the European Medicines Agency (EMA), the pharmaceutical industry, non-profit foundations, patient representatives and academia [12]. Some recommendations have since been drawn up to enhance the use of ADs [11, 12]. Although this has been a significant milestone, the NIH workshop did not explore the perceptions and attitudes towards ADs of key stakeholders directly involved in the day-to-day conduct of clinical trials. Furthermore, some of the NIH findings may not be directly extrapolated to the UK setting, due to differences such as public funding and clinical trials infrastructure, capacity issues and underlying perceptions.

Little research has been undertaken to explore the use of ADs in the publicly funded confirmatory setting, particularly in the UK. In 2012, Morgan and colleagues [3] investigated the use of ADs and associated perceptions of barriers in the private and academic sector through a survey. The authors found change management, regulatory acceptance, lack of education and extra time and resources required for planning as major perceived barriers. Jaki [13] also investigated the use of ADs and Bayesian methods in early phase trials through a cross-sectional survey of registered UK Clinical Trials Units (CTUs), predominantly surveying statisticians. The poor application of these methods was attributed to five key barriers: lack of software, clinical investigators insisting on preferred methods, lack of expertise, inadequate funding structure and time required for trial design. These researches demonstrated the existence of barriers impeding the use of ADs. We have endeavoured to fill the gap in previous research [2, 3, 8, 9, 11–14] by incorporating nested qualitative interviews of key stakeholders with diverse roles in clinical trials research with a focus on publicly funded confirmatory trials prior to subsequent related surveys to be reported elsewhere.

This study is motivated by the belief that further related research and discussions are needed in the UK publicly funded confirmatory setting. Understanding perceptions towards ADs by researchers and decision-makers is key to unlocking potential benefits of ADs in this setting. We therefore aimed to explore key stakeholders’ experiences, perceptions and attitudes towards ADs in publicly funded confirmatory trials and their views on barriers and facilitators to the use of ADs. We believe our findings will inform researchers and decision-makers on key issues, in order to facilitate their preparedness to utilise acceptable ADs in publicly funded confirmatory trials where appropriate.

## Methods

### Study design and setting

This study valued the importance of understanding obstacles to AD use from the point of view of key stakeholders’ experiences, perceptions and attitudes, in order to generate facilitators to unlock barriers to appropriate use. This approach, which explores views, meaning and context, can be viewed within the phenomenological paradigm [15]. Hence, we conducted cross-sectional, in-depth, semi-structured, one-to-one qualitative interviews of key stakeholders involved in clinical trials research [16]. This approach encouraged participants to talk about pertinent issues about ADs through the use of open-ended questions. Some of these questions were *a priori*-designed based on topics from previous literature [7–9, 11, 14], and others were informed by researcher-driven hypotheses. Although we paid attention to the UK publicly funded setting, a cross-sector approach was undertaken by including participants with private sector experiences in order to explore diverse experiences, perceptions and attitudes. In particular, we purposively sought expertise in the private sector due to a perceived greater experience of ADs [2]. In addition, four international participants were included in our sample in response to advice given by some participants during the interviews. We conducted interviews by telephone or through face-to-face conversations based on feasibility and the need to reach out to a wider geographical area of participants of interest.

### Sample size

Most qualitative studies base their sample size on reaching data saturation, which is unknown in advance because it depends on various factors such as: the scope and nature of the research subject, study design and resources available [17–20]. Some authors recommend up to 10 homogeneous interviews for phenomenological research [19]. Bearing this and time constraints in mind, we intended to recruit six to eight participants per expertise category, yielding a minimum of 20 participants depending on the degree of overlap in expertise. We also adapted our sampling in some expertise categories guided by richness of information from previous interviews and the need for further exploration of certain phenomena. Overlapping of participant roles afforded an opportunity to explore wider views and experiences with a smaller sample.

### Selection of participants

We purposively selected participants in a consecutive manner following informed consent agreement if they met the desired core duties and responsibilities in trials research. We adopted this cross-disciplinary approach to optimise maximum variation to capture diverse views and experiences [21]. Core expertise for purposive sampling were UK CTU leaders (directors or deputy directors), public funding panel and board members (chairs or vice chairs including other ordinary members), Independent Data Monitoring Committee (IDMC) members, regulators, statisticians, health economists and chief investigators. We sent an invitation letter with an information sheet to target participants using various platforms; mass emailing to specialist network groups including the UK CRC Registered CTU Network [22] and the MRC Network of Hubs for Trial Methodology Research [23]; and personalised emails to referred contacts and hard to reach groups, such as private sector, regulators and public funding panel and board members.

We phrased the invitation letter to emphasise that participants would be eligible to participate regardless of their underlying experiences, perceptions and attitudes towards ADs in order to minimise potential responder bias due to oversampling of participants likely to express positive views. We asked responders to complete a short questionnaire detailing their demographic characteristics and key expertise and to return it with their signed informed consent form. We then sequentially selected participants until reaching the desired target sample size. We used interview guides tailored for participants’ expertise to prompt questions (see Additional file 1). We undertook five internal pilot interviews, four of which were face-to-face to test the appropriateness of the interview guides, prompts and interview duration. On completion of the interviews, we gave participants an option to verify their interview transcript and also to say anything relevant about ADs that they felt was not covered but worth contributing.

The lead author (MD) conducted the interviews, which were audio recorded and verbatim transcribed by experienced in-house transcribers. A favourable ethical opinion (0676) was received from the Research Ethics Committee of the School of Health and Related Research at the University of Sheffield, and all interviews were conducted with signed informed consent.

### Analysis and reporting

Data were entered into NVivo10 [24], which was used to manage and organise the data analysis process. We employed the framework method [25, 26] to structure the analytical process, which includes the following key stages; familiarisation and annotation of transcripts, identifying a thematic framework [27], indexing, charting, mapping, and interpretation [28–30]. Mapping helped to identify relationships and clusters around themes, thereby facilitating understanding, communication and interpretation. Themes captured what is most important from the data in understanding views and experiences concerning the use of ADs. We adapted taxonomies developed and used by other authors in the field of evidence-based practice to classify barriers to the uptake of ADs into micro- and macro-level domains pertinent to key stakeholders at the individual and organisational level [31–33]. Some potential facilitators to perceived barriers are also presented. We paid attention to emerging themes and contrasted these with the existing literature. Supplementary interview data and case studies to support themes are provided (see Additional file 2). We utilised the COREQ checklist to guide the conduct, analysis and reporting of this study [34].

## Results

### Description of participants

In total, 27 participants were interviewed between March and August 2014, predominantly based on a sampling frame of 45 registered UK CTUs (2012/2013). Previous AD experience of participants is shown in Table 1. One health economist agreed to be interviewed. Reasons for not taking part among 17 health economists who were directly invited were unfamiliarity with ADs (*n* = 5), busy schedule (*n* = 1), non-response (*n* = 10) and willing but incompatible schedule (*n* = 1).

**Table 1:** Characteristics and demographics of interviewed participants

Variable	Scoring	Total
		(*N* = 27)
Sex	Male	17(63 %)
	Female	10(37 %)
Age group (years)	>30 to 35	4(15 %)
	>35 to 40	2(7 %)
	>40 to 45	8(30 %)
	>45 to 50	4(15 %)
	>50 to 55	4(15 %)
	>55	5(19 %)
Academic qualifications	MSc/MA or equivalent	3(11 %)
	PhD/DPhil/DSc or equivalent	21(78 %)
	Other	3(11 %)
Trials experience (years)	0 to 2	1(4 %)
	>2 to 5	1(4 %)
	>5 to 10	2(7 %)
	>10 to 15	6(22 %)
	>15	17(63 %)
Current employment sector	Private	4(15 %)
	Public^a^	22(81 %)
	Both private and public	1(4 %)
Mode of interview	Face-to-face	7(26 %)
	Telephone	17(63 %)
	Skype telephone call	1(4 %)
	Skype video call	2(7 %)
Location	UK	23(85 %)
	International	4(15 %)

^a^Six participants had previous private sector experiences. Participants’ diverse previous AD experiences: none (*n* = 9), of which six expressed interest in ADs; during planning only (*n* = 6); during planning and conduct, either in early or confirmatory phase or both (*n* = 8); statistical regulatory assessment (*n* = 4)

Interviews were conducted by telephone (*n* = 17), face-to-face (*n* = 7), skype video call (*n* = 2) and skype telephone call (*n* = 1). Median duration (IQR) of interviews was 31 (26 to 38) minutes ranging from 13 to 51. Participants’ characteristics, demographics and diverse overlapping primary duties and responsibilities in clinical trials research are displayed in Tables 1 and 2.

**Table 2:**
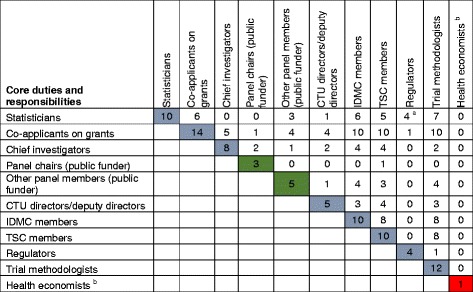
Overlap of core duties and responsibilities of 27 participants in clinical trials research

^a^ Duties and responsibilities during the interview fit in with statistical regulatory assessments although not indicated as statisticians on the baseline form

^b^ Member of a national health economics appraisal board

Interpretation: For instance (row 1), we interviewed 10 Statisticians with other overlapping roles: six had been co-applicants on research grants, three served on public funding boards or panels, one served as a UK CTU leader, six were IDMC and five TSC members, and so forth

### Perceived value of ADs and opportunity for use in confirmatory trials

#### Perceived advantages of ADs

Participants stated potential advantages of ADs, which can be broadly classified into three categories: ethical benefits to patients, improving design efficiency to answer research question(s), and value for money. These perceived advantages are summarised with supporting data in Table 3 and Additional file 2.

**Table 3:** Characterisation of potential benefits to patients, clinical trials and funders

Thematic area	Characterisation of potential benefits	Some supporting quotations
Ethics: patient benefit	Early stopping of trials as soon as there is sufficient evidence to answer the research question(s) means:	‘It really depends on the type of AD so if you have a GSD (Group Sequential Design) then of course you can stop early for futility or overwhelming effect and this clearly has many ethical and financial advantages. So for futility stopping –if it doesn’t work you can stop early on and the patients don’t get exposed to a drug that does not work or if you have overwhelming effect that is also very positive you can move on with the development of your drug and you don’t have to finish the whole trial’. (QL11 Statistician)
	▪ Minimisation of unnecessary over recruitment of research patients	
	▪ Acceleration of the evaluation of interventions, approval and commissioning into practice. Thus, patients receive effective interventions quicker	
		‘…from a patient point of view the sooner that if there is a new intervention that is really effective then we want to get that into NHS (National Health Service) practice, equally if it is dangerous or if there is anything that we shouldn’t be using then we would want to get that out and into guidelines and NHS practice as much as possible’. (QL01 CTU Deputy Director, Proposal Developer)
	▪ Minimisation of the exposure of patients to potentially ineffective and/or unsafe interventions	
	▪ Patients are likely to be allocated to interventions to which they have a higher chance to respond better; vital in critically ill patients	‘When you certainly have a limited patient pool like orphan disease implications where you know you’re not going to be able to actually recruit sufficient patients for a full Phase 2/3 traditional development programme and we accept and understand that, as regulators and industry, you’re offered appropriate incentives under orphan designation in the EU (European Union) and US (United States). So, there’s undoubtedly a challenge - an opportunity to maximise the best use of patients. I've once actually seen a combined Phase 1/2/3 study, all in one go’. (QL16 Regulator)
	▪ Identification of group of patients who are most likely to respond to the intervention [61, 62]	
Efficiency in trial design	▪ Mitigating the risk of making wrong design assumptions	
		‘One of the things that I always tend to tell people is that ADs will make you address the objectives of interest enabling you to make the right decisions earlier rather than later, for example, the biggest opportunity is stopping poor drugs early, most of our drugs fail, 90 % of the drugs that we start developing in phase 1 never get to the full registration, we should be killing those drugs as early as possible and ADs allow you to do that, whether it is in phase 2 or 3 there is always that opportunity to stop early for futility’. (QL15 Statistician)
	▪ Allows simultaneous testing of multiple interventions from a competing list with option for dropping inferior interventions in a single trial rather than multiple series of two-arm trials [46]	
	▪ Allows the answering of research questions quicker to expedite decision making	
		‘The main advantages of ADs accrue to funders because funders are not paying for essentially redundant data and ethically I think there is a benefit to patients because clearly we don’t want to be recruiting patients to trials when there is no significant potential of that trial and additional data giving you any new information’. (QL04 Chief Investigator, Vice Chair - Public Funder)
	▪ Efficient use of a limited patient pool, particularly in rare or orphan disease implications	
	▪ Efficient use of a finite pool of clinical chief investigators	
Value for money for funders/sponsors	▪ Avoiding pursuing the lost cause when trials are stopped early for futility	
	▪ Efficient use of available research resources. Stopping trials early means resources are reallocated to other promising or priority areas	

### Perceived therapeutic areas of opportunity to use ADs

In principle, most participants acknowledged that ADs could be applicable across a wide spectrum of therapeutic areas (see Additional file 2). However, some participants also believed ADs may be more appealing or appropriate for certain health conditions or populations, due to factors such as severity of the health condition, availability of standard care options and limitations of standard methods (such as in small populations).

Some participants mentioned the potential value of ADs in areas including, but not limited to oncology; emergency medicine; and respiratory, cardiovascular, infectious and rare or orphan diseases. Participants stated that the nature of the clinical outcome(s) of interest and study intervention may also influence relevance of ADs. A case study was shared on how ADs could be of value in evaluating interventions during outbreaks of rapidly evolving and fatal pandemics such as influenza or Ebola, due to the severity of the conditions, coupled with the need for urgent clinical decision-making by policymakers (see Additional file 2, Case Study A). Most importantly, participants stated that it is imperative that the proposed AD is fit for the purpose of robustly answering the research question(s) accompanied by a clear rationale.

### Perceived types of ADs with potential in confirmatory setting

Participants mentioned that the following types of ADs have useful potential within the confirmatory setting, which are also reflected in the literature [1, 2, 8, 9, 35–38]. However, the receptiveness towards these ADs by the research community and policymakers varied considerably depending, for instance, on the type and scope of the adaptation. These types include the following:

1. Sample size re-estimation (SSR) conducted either in a blinded or unblinded manner.
2. Futility assessment based on stochastic curtailment (such as conditional power)
3. Standard group sequential design (GSD) allowing for early stopping (such as for futility and/or safety and/or efficacy and/or non-inferiority).
4. Strictly phase 3 multi-arm multi-stage design (MAMS) with treatment selection allowing for dropping of inferior or futile treatments and/or early trial stopping.
5. Operational or inferential seamless 2/3 design with treatment selection in phase 2.
6. Response adaptive randomisation (strictly based on primary outcome data).

### Public funders’ perceived change in attitudes towards ADs

We inferred a paradigm shift in attitudes towards ADs by public funders, mainly motivated by value for money and the desire to make use of the public purse more efficiently. Funders expressed a clear positive will and receptiveness to fund adaptive trials and to encourage researchers to utilise ADs, whenever appropriate, to answer the research question(s). This view, supported by various initiatives relating to ADs funding opportunities (such as training fellowships and grant calls) [39–41], was acknowledged and welcomed by researchers.

‘I think generally speaking we are receptive to those ideas (of ADs) and in fact we, at [organisation] have held our own workshops on ADs last year or the year before in order to try and promote more use of ADs providing they are appropriate of course. So I think 10 years ago our attitudes were more towards traditional parallel group, it was a sort of traditional well-known pathway but I think now our modern thinking is that we welcome ADs when appropriate and it is very much for the applicants to make the case for why they want maybe four arms with interim analyses for dropping arms …’ (QL35 Chair - Public Funder)

### Regulatory receptiveness and improving awareness and experiences

In principle, there appeared to be regulatory receptiveness towards the concept of ADs, but this was conditional on strong caveats, particularly in confirmatory trials on measures such as minimisation of operational bias during the conduct to preserve trial credibility and integrity, control of type I error, and use of appropriate statistical inference. These caveats and considerations are highlighted in regulatory guidance and reflection papers specific to drugs and biologics [42–44]. We inferred that regulatory awareness and experience is growing, particularly among statistical assessors due to the increasing number of AD-related scientific advice consultations and applications by researchers; especially on SSR, futility assessment and GSD trials, as also reflected in recent literature [14, 45].

‘I haven’t got the figures in front of me and I wouldn’t know how to get them but you see a lot more of them at the moment in the scientific advice arena, when people are coming saying ‘this is what we are going to do, what do you think?’ … I get a lot of them starting and not so many of them finished yet’. (QL19 Regulator, regulatory assessment experience)

Regulators advised researchers to engage them through scientific advice meetings and to adhere to their guidance when considering appropriate ADs from trial planning to the end.

### Cross-disciplinary interest and positive clinical will

Most participants expressed widespread growing interest towards ADs even through there are existing concerns.

‘I guess there are a lot of concerns about them and so that’s perhaps why they’re not taken up so much. But it is interesting to see that there’s a lot more interest in the past few years and so maybe that is changing’. (QL26 Statistician, design and conduct experience)

‘… influential bodies like the FDA are now embracing ADs and there is probably an increasing number of ADs that are being utilised and will come through and report over the next 2/3/4 years …’ (QL21 Chief Investigator, design experience)

We also inferred a positive will by clinical investigators contradicting previous related findings in early phase trials, suggesting that clinical investigators insist on application of certain methods [13].

‘We definitely have an interest in advancing new methods in the field of sepsis and in particular there is probably room for improving clinical trial design and that is the focus of our group (ADs methods)’. (QL22 Chief Investigator, design experience)

Most importantly, the positive desire expressed by clinical investigators appears to depend mostly on how trialists market ADs to them and the availability of support during trial planning and conduct.

‘I think generally once you have explained it (AD concept), and you have said that it will be a very big expensive trial if we did it fully powered for as long as it would take, but say that it can be broken down to give different options to the funder for shorter time periods, and less cost, then they can see the advantages to it. … if we are happy to do it and design it and write that section up for them they will take it on*’.* (QL01 CTU Deputy Director, Proposal Developer, design experience)

‘Sometimes you need to sell it to them to get them to see its positives and advantages and in terms of the extra complication it takes to implement them’. (QL07 Statistician, design and conduct experience)

Cross-disciplinary interest appears to be influenced by the desire to improve design efficiency to answer clinical research questions, address ethical aspects and maximise value for money in research (Table 3).

‘… (ADs) makes a lot of sense from my point of view and in terms of optimising the design and feasibility of the study to address the particular research question. I think it is important that statisticians and clinicians discuss thoroughly the options that are available in clinical trial design to agree the best proposal because each will have particular insights with regard to how to address a research question and so communication is really essential’. (QL24 Chief Investigator, design experience)

‘…there is a lot of interest in them from a funder’s point of view, in that particularly difficulties in recruitment, when it has taken a long time to recruit for trials when recruitment is not up to its expected levels, it is very helpful to be able to have a design that allows you to have multiple looks at the data and to potentially stop early’. (QL04 Chief Investigator, Vice Chair - Public Funder, design experience)

### Perceived potential barriers to the use of ADs in confirmatory trials

#### Cross-disciplinary lack of awareness and understanding

Some participants viewed the widespread lack of awareness and understanding of different types of ADs, circumstances when ADs are appropriate, and implementation resources, as barriers to appropriate use. Consequently, some participants believed that there are missed opportunities and underutilisation of ADs when appropriate in some trials. Some expressed worry that misunderstanding of when ADs are appropriate may lead to misuse, due to their growing prominence - even in certain circumstances when ADs are not superior to traditional fixed sample size designs.

### Confusion over what is meant by an AD

One resonant finding we inferred is the potential for misunderstanding of what is meant by the term AD and its acceptable scope in the confirmatory phase. Most participants acknowledged a broadening of the scope of what is considered to be an AD in recent years. Consequently, the term is often loosely defined, but with broad contextual meaning prone to misinterpretation leading to confusion among researchers.

‘I would say, over the last three years, I’ve become aware of (the) detail of ADs. Prior to that, it was a sort of loosely bandied term … I could be in a room and everybody thinks they’re talking about the same thing and they’re talking about very different things’. (QL8 CTU Director, no experience)

‘So I am generally in favour (of ADs); however, convincing the community of that takes some work, so a big threat for ADs is just that it’s a cutesy word that means different things to different people, there’s misinformation about it and there are some existing biases in the community and so there really needs to be a lot of education’. (QL22 Chief Investigator, design experience)

Although this confusion has been partly addressed from a regulatory and industry perspective [8, 42, 43], some participants viewed it as a current problem in the public sector, where most study interventions do not require regulatory approval beyond standard ethics.

### Cross-disciplinary conservatism

Most participants viewed cross-disciplinary conservatism as one of the major barriers to the usage of ADs, particularly in the confirmatory phase. This conservatism depended on: Most importantly, we inferred a complex multifaceted degree of conservatism, which appears to be influenced by many factors. Table 4 summarises subthemes inferred to influence conservatism and negative attitudes towards ADs.

**Table 4:** Inductive themes perceived to be linked to conservatism

Stakeholder	Secondary theme associated with conservatism	Contributors linked to secondary theme
Cross-disciplinary	▪ Unfamiliarity and lack of understanding	
	▪ Fear of introducing operational bias during conduct and compromising the trial	
	▪ Concern about the robustness of ADs in decision-making	▪ Fear of making wrong decisions
		▪ Concerns about premature early stopping of trials
		▪ Concern that the research community may struggle or be reluctant to accept the findings from an adaptive trial
		▪ Contrived general perception by journal editors and reviewers that early trial stopping is a failure
		▪ Impact of early trial stopping on other secondary but important objectives
	▪ Research teams being more comfortable with traditional fixed designs than ADs	▪ Sticking to what we know best and fear of venturing into the unknown
		▪ Lack of knowledge and experience
		▪ Generation effect - more senior trialists being sceptical of change from what they know best and perceive as standard
		▪ Perceived operational and statistical complexities during planning and implementation
Regulators	▪ Buy in reluctance in confirmatory setting	▪ Lack of understanding of the inferential and regulatory price to pay by using an AD
		▪ Fear of lowering the level of evidence
		▪ Fear of making wrong decisions that may taint their reputation in the future (for instance, approving a drug that will subsequently be proved to be unsafe or ineffective)
		▪ Limited experiences in the assessment and approval of ADs
Statisticians	▪ Negative attitude towards ADs among some influential statistical community	▪ Generation effect - more senior researchers being sceptical of change from what they know best and perceive as standard
Private and public funders	▪ Reluctant to fund potential high risk high value research projects with huge uncertainty	▪ Uncertainty around the actual sample size, duration and actual cost of the trial
		▪ Inadequate description of variable costs, decision-making criteria and time frames on grant applications (public funders)
	▪ Limited commissioning and funding experiences, especially among public funders	
		▪ Difficulties in drawing up flexible employment contracts (public funders)
		▪ Limited number of AD grant proposals being submitted by researchers for consideration (public funders)
	▪ Negative attitudes towards ADs among some public funding panel members	▪ Lack of familiarity
IDMC and TSC members	▪ Perceived negative attitudes towards multiple examinations of the trial data	▪ Lack of familiarity and understanding
	▪ Reluctant to stop trials early unless for safety reasons

1. Trial phase and nature of research objective(s),
2. Health condition or study population and nature of intervention under consideration,
3. Rationale put forward and completeness in description of the proposed AD(s),
4. Type and scope of proposed AD, the availability of well-established methods for statistical inference, and perceptions towards that AD by policymakers,
5. Perceived complexities associated with the AD and impact on implementation, potential introduction of operational bias during conduct, and interpretation of the findings, and
6. Underlying familiarity and understanding of the proposed AD.

Most participants stated that there is limited scope for ADs in confirmatory trials, due to the definitive nature of research objectives, with direct influence on policymakers’ decisions to approve new interventions into clinical practice. Moreover, some participants strongly advised against conducting too many adaptations in confirmatory trials, citing difficulties in the interpretation of the findings, which may undermine trial credibility.

‘… people should be cautious I guess in trying to do too much and having too many adaptations … We must still make sure we have that body of confirmatory evidence, so I think there might be a place in phase 3 for ADs, but only sort of minimal adaptations. We should sort of keep things under control in that particular setting …’ (QL19 Statistician, regulatory assessment experience)

Insufficient description of proposed ADs, with their statistical and operational characteristics supported with evidence (such as from simulation work or established references) was viewed to influence conservatism towards ADs. This view also reflected the FDA’s regulatory guidance position, which classified well-understood from less-understood ADs [8, 43].

We found that the MAMS AD attracted cross-disciplinary attention, particularly from policymakers, citing efficiency and value for money in testing multiple interventions in a single trial, allowing for dropping futile arms, as opposed to conducting multiple series of independent two arms trials [38, 46].

‘In terms of the multi-arm trials I’m much more comfortable now with the idea of maybe setting out, even on a phase 3 trial, with 4 or 5 potential interventions and dropping the ones that look least promising’. (QL14 Statistician, no experience)

### Lack of knowledge and experience

Cross-disciplinary lack of knowledge and experience of ADs was perceived as a major barrier. Most participants viewed this to be intertwined with insufficient access to case studies to facilitate practical training, to raise the awareness of benefits and an understanding of when ADs are appropriate, and learning about barriers and facilitators to successful implementation. Certain participants raised concerns about deficiencies in current training approaches, which they viewed as more oriented towards statistical methodology rather than translational practical training. In addition, weaknesses in some current academic graduate training curricula, which do not tend to incorporate ADs as alternative designs, were articulated.

‘… the main challenge … I think it is a bit broader - is the lack of experience and knowledge within the bio-statistics community. There is a lack of understanding of adaptive methods, a lack of understanding of the opportunities, you know and a lack of familiarity’. (QL12 Clinical Research Leader, Trial Methodologist, design and conduct experience)

A number of participants conveyed a lack of familiarity and knowledge of alternative ethical and efficient designs among ethics and scientific review board members, which may hamper their ability to adequately review grant proposals. The lack of capacity and competency of peer reviewers of AD grant proposals in the public sector was also a perceived barrier. Similar concerns were also reflected in the United States [11, 12].

### Degree of statistical and operational complexity

#### Amount of work and effort required and marketing of ADs to key stakeholders

Most participants stated that ADs, in general, require additional work and effort from a statistical and operational perspective compared to traditional fixed sample size designs during planning and implementation. Consideration of operational feasibility - how implementation of the AD is going to work in practice - was viewed to be vital. Operational feasibility encompasses aspects such as logistics, administration, resources, primary endpoint relative to the expected recruitment rate, implications of trial governance processes and collaborating sites, and intervention delivery [14]. However, the level of statistical and operational challenges depends on the nature of the proposed AD and tends to increase with its complexity. A number of participants believed that more time and effort (depending on the type of AD) is required in marketing the rationale for the proposed AD to key stakeholders (such as funders, regulators and clinical collaborators) and in planning, compared to traditional fixed sample size designs.

#### Statistical simulation work at the design stage

Some participants mentioned that ADs require more effort and time (depending on the complexity of the AD) to conduct adequate simulation work under various scenarios, and to understand the statistical properties of the design and its implications on decision-making [35]. Some of our interviewees mentioned the concern about inadequate simulation work and its consequences on statistical properties and decision-making. Regulators raised similar concerns about a response adaptive randomisation case study, concerning whether the simulation scenarios covered the entire domain of the desired sample space to guarantee control of the type I error (see Additional file 2, Case Study C). Some participants identified the need for applied training of statisticians on how to undertake adequate simulation work of ADs.

#### Robust data management infrastructure

Some participants viewed data management and related logistical challenges as potential barriers due to the need to minimise operational bias in the conduct of ADs and to provide clean, robust data to inform the adaptation process. The following considerations were raised:

1. Compatibility of data management infrastructure with collaborators;
2. Real-time data capturing, cleaning and processing. An example of a successful multi-centre case study, which used tablet computers for real time electronic data capturing in an African-based trial setting was shared (see Additional file 2, Case Study B);
3. Turnaround time of data management processes to inform adaptation; and
4. Systems, processes and procedures supported with audit trails to minimise potential operational bias encompassing the sort of information that should be disclosed and to whom, how the information should be transferred, and firewalls and clarity on who is doing what.

#### Confidentiality and implications of ADs on IDMC duties and responsibilities

The need to maintain confidentiality by the IDMC during communication and execution of their duties supported with documentation was viewed as paramount. It was advised that the training of and discussions with IDMC members prior to trial commencement regarding the proposed AD; related decision-making criteria; execution of their duties as guided by formalised documents, such as a charter [47]; communication protocol; and clarification on related issues are essential. Some participants stated that ADs, depending on complexity, may require more effort, time and expertise for the IDMCs in understanding the design, its decision rules and execution.

#### Additional statistical considerations

The availability of in-house statistical expertise supported with quality control, validated software or user written statistical codes to execute the AD, and delivery time of results to inform interim decision-making were some of the statistical obstacles raised. However, these depend on the type of proposed ADs. An experienced statistician shared a case study where they adapted the methods from another clinical area using a different endpoint to suit their research question but with additional statistical work and time commitment (see Additional file 2, Case Study B).

### Concerns around trial credibility and integrity

Most participants expressed strong preference for planned ADs, with decision rules clearly pre-specified at the design stage: this facilitates adequate understanding of the design’s statistical properties through simulation and enhances proper planning. Most importantly, pre-planning of ADs is a regulatory necessity to safeguard trial credibility, integrity and validity, especially in the confirmatory setting [10, 42]. A resonant view was that ADs are not a remedy for poor planning, and most participants were concerned about *ad hoc* (unplanned) adaptations, which they viewed with great suspicion, regarding such activity as cherry-picking and potentially hiding negative findings to advance the hidden personal agendas of some researchers. Preference for ‘prospectively-planned adaptation’ or the ‘adaptation by design’ concept, particularly in the confirmatory phase, is reinforced in the literature and regulatory guidance [8, 9, 35, 43].

Fear of compromising the trial by potential introduction of bias during its conduct and potential population drift during adaptation were viewed as major concerns, which could be due to dissemination of the interim results [8, 35]. The need for safeguards and firewalls to minimise leaking of interim results, with clear processes, procedures and documentation with audit trails was reinforced [10, 14]. These shared views are in agreement with some of the considerations highlighted in the EMA reflection paper [42] and the FDA guidance document [43].

‘I think it (ADs) will always raise an element of suspicion if there have been some decisions made along the way that have been data driven. And the key thing is just to have all the documentation in place; it has to be set out precisely in the protocol how it will be done and you need the right mechanisms in terms of the monitoring committee or steering committee makes the decision and make sure you comply with all the mechanisms. I mean it’s like GCP (Good Clinical Practice); it’s not enough to do the right thing, you’ve actually got to be able to prove you’ve done the right thing… with adaptive trials it’s that much harder to prove that you’ve done it legitimately. So you’ve got to be very careful about the process and got to be able to demonstrate through documentation that you have followed true process’. (QL14 Statistician, no experience)

Although most participants acknowledged routine monitoring as part of every trial, some expressed concern about the lack of understanding of the impact of *ad hoc* changes on the statistical properties of the design, introduction of bias, interpretation and credibility of the findings [35]. They also highlighted the need for some minimal flexibility as part of routine monitoring in case of unexpected events within a planned AD framework. Anticipation of possible scenarios as much as possible at the planning stage was viewed as imperative.

### Concerns around trial validity

A number of statisticians and regulators expressed anxiety about the use of appropriate statistical inference following an AD, arguing that little attention is paid by researchers to the impact on trial results (estimates of treatment effects, confidence intervals (CIs) and P-values). However, they seemed to acknowledge that the awareness regarding control of the type I error rate has improved. More so, some participants highlighted the need for adequate transparency in the conduct and reporting of ADs, and opinion seems divided on whether the current CONSORT guidance is fit for purpose in the case of ADs.

### Public sector perspective

#### Worry about impact of ADs on research staff employment contracts

Some participants stated that the existing public funding models for fixed trial designs create financial uncertainty for research staff employment contracts when trials are stopped early [13]. Consequently, there is nervousness among some UK CTU directors to support certain ADs with options for early stopping. However, some participants stated that this problem is not unique to ADs because some fixed designed trials are stopped early, mainly due to poor recruitment [48, 49]. In contrast, they also stated that design flexibility is somewhat inevitable, due to a paradigm shift by some UK public funders towards risk assessment within an internal pilot framework, with associated staggered research contracts. In addition to the UK CTU reputation and experience, some participants viewed that concerns about the impact of the funding model on staff contracts depends on factors such as the following:

1. Type of AD proposed - some ADs such as SSR and MAMS are less likely to be affected.
2. Size of the research group and trial portfolio - large UK CTUs can more easily reassign staff to other trials in the pipeline when an AD is stopped early.
3. Remit of the public funder - some have more flexible funding models than others.

‘… Because of the size of the trials unit there are many trials that are taking place so we look very closely at people’s contracts and what studies are taking place, it’s not just based on 1 study. We have a lot of different trials at the trials unit so the infrastructure allows for -if the trial stops early then they would be able to work on another trial. So it is not driven by the fact that the contracts or by whether or not it would stop early on this particular trial because of the other trials taking place requiring statistical, trial management, data management support’. (QL27 Statistician, design and conduct)

Many participants acknowledged the need for public funders to draw up standardised, flexible funding agreements compatible with key research partners: UK CTUs, Universities, sites and UK CRN [50]. Some suggested this could be achieved through modification of the current staggered research contracts employed for studies with internal pilots.

#### Lack of capacity within UK CTUs and time limitation

Most participants stated that there is a lack of expertise and capacity, particularly a dearth of statisticians and proposal developers with knowledge to support complex ADs. However, they acknowledged that capacity and expertise varies across UK CTUs. The majority of participants mentioned that they have limited time to support design work of complex ADs - citing the extra work required against pressure to deliver on competing priorities based on simpler, traditional, fixed sample size designs.

‘One is just the lack of expertise within the unit, so it is easier when you are very busy to put forward a design you know rather than one you don’t. It is also easier because if you put forward a design that does not look the same to clinicians who expect straightforward designs you have to be very confident in that design to be able to convince them to some extent’. (QL9 CTU Director, Statistician, design experience)

#### Lack of bridge funding for UK CTUs to support planning

Some UK CTU directors voiced concern about the lack of a business case [13] - citing the amount of time required to support the design of complex ADs, which is unpaid for, betting on uncertain future success of grant applications. Similar concerns have been raised in the US publicly funded setting [11, 12]. UK CTU directors called for funding opportunities in the form of developmental grants to support adequate design work of complex ADs, conditional on meeting research and funding priorities.

‘I think for some of the really complex ADs it would be good if there was availability to go for some small trial development grants so that you could say ‘look this is a convincing clinical question, we think it should be approximately this sort of design but actually we need £20,000 or whatever to properly work it up and design it’ and that type of trial development grant I think would help unlock some of that’. (QL09 CTU Director, Statistician, design experience)

Although this was acknowledged, a funder expressed a contrasting view; citing that in the UK, bridge funding is partly addressed through the NIHR infrastructure support funding accessible to over 25 accredited UK CTUs on a rolling contract basis [51]. However, UK CTU directors’ views appear to suggest that this funding is insufficient, and there is high risk attached to supporting the design work of complex ADs. Funders suggested that researchers may need to consider applying for small grants within the remit of other NIHR funding streams to support developmental work of ADs.

‘Typically for complex ADs then you have to do quite a lot of modelling -that could take 12 or 18 months. Ideally, there should be grants to cover that early development work. Yes, I have sympathy to the idea that there needs to be additional funding but on the other hand I suppose all work that CTUs do prior to a trial application is done at risk. When I was CTU director, typically you are talking about 2 years work before you applied to do a definitive trial. I could say there ought to be more grants to help with all of that and the reality is that we in [organisation] in a sense do pay that upfront because we have a scheme whereby we support CTUs, we give them £250,000 per year if you like, like a front loaded loan, which they use to buy core staff in order to develop new projects. So in a way I think we are doing it already’. (QL25 Chair - Public Funder)

#### Limitations of the grant application process

In the case of complex ADs, some participants suggested the need to increase the proposal development time prior to submission deadlines to give researchers adequate planning time, particularly for commissioned calls. Some authors suggested a minimum of 3 months for design and planning for ADs [14]. Moreover, a slight modification to the grant application form depending on the funding remit was suggested, to give more space for researchers to describe the rationale, design and its properties, decision scenarios and variable costs adequately.

‘From a practical point of view when you are designing adaptive trials there is more work involved for the application in planning the trial and working out the timelines … you have to do it for a number of different scenarios. So the work involved in that is more from the trialist and statistician’s point of view, the statistician has to do various modelling and look at different scenarios and we have to do all of the different planning and you are usually on a fairly tight deadline for applications because of the way that NIHR funding works so if you only have 6 weeks to work with the team, trying to fit in time to do lots of different scenarios can be quite tricky and can make it more difficult’. (QL01 CTU Deputy Director, Proposal Developer, design experience)

‘There is not an existing section in grant submissions that says ‘if you are doing an adaptive trial design please provide the following information’, so I just don’t know that it’s well organised yet and that could be a good thing or a bad thing …’ (QL22 Chief Investigator, design experience)

## Discussion

### Contributions of this study and implications for practice and future research

We found the following cross-sector perceived barriers to the use of ADs in confirmatory trials among the stakeholders we interviewed:

1. Lack of practical knowledge and applied training coupled with insufficient access to case studies to facilitate practical learning.
2. Time constraints to support planning relative to other competing priorities based on traditional designs.
3. Lack of awareness of opportunities about when ADs are appropriate in conjunction with the lack of understanding of their acceptable scope in confirmatory trials.
4. Statistical and operational complexities during planning and implementation of ADs.
5. A cross-disciplinary degree of conservatism influenced by various factors.

Specific to the public sector the barriers included lack of bridge funding accessible to UK CTUs to support the design work of complex Ads, difficulties in marketing ADs to collaborators, anxiety about the impact of early trial stopping on full-time researcher employment contracts and lack of capacity to support ADs. Some of these barriers have already been previously reported [2, 5, 11, 13, 14].

Practical education tailored to trialists is paramount to address the lack of practical knowledge. Activities such as educational seminars or webinars and practice-oriented workshops can facilitate translational knowledge sharing. The content of such activities should cover the practical, statistical and logistical issues that need to be addressed in planning and conduct of adaptive trials with the aid of case studies where possible.

We strongly encourage accessible publication of ‘successful’ and ‘unsuccessful’ case studies of ADs previously undertaken, addressing aspects beyond the primary results, such as practical barriers and facilitators, which will complement the educational resources. These should include positive and negative lessons learnt to help the design and conduct of future adaptive trials. Adequate reporting and indexing of these AD-related publications of case studies is important. We are now witnessing such publications in the literature [52–56].

The establishment of small design developmental grants accessible to UK CTUs could encourage trialists wishing to undertake time-consuming and complex ADs, which are efficient to address research questions. Such small grants could be a collaborative initiative among public funders such as the NIHR and MRC. There is an issue that needs to be addressed of funding for statistical AD design work being granted before the merits of the scientific clinical question have been fully addressed. This may entail trialists going through a multi-stage grant application process. An initial ‘Outline’ stage could encompass putting forward the research question, justifying its importance, design rationale, explaining the gap in the design requiring further work, and time and resources required to undertake such work. Trialists could then be sign-posted to apply for small grants to develop the design conditional on the ‘Outline’ proposal meeting research and funding priorities. Further funding of the main trial could then be available conditionally, subject to the outcome of the design work. In addition, there should be mandatory open access publication of the initial stage design-related outputs such as statistical software or implementation codes to help the design of future similar studies.

We found evidence of a complex, multifaceted cross-disciplinary degree of conservatism, which appears to influence perceptions and attitudes towards use and acceptability of ADs among our study participants. We uncovered some of the factors influencing this conservatism, thereby aiding our understanding to address barriers to use and acceptability of ADs. For instance, concerns that the research community, clinical community and policymakers may struggle to accept findings from ADs to influence policy underscores the strong need for methodological assurances and effective communication regarding robustness in decision-making. We believe adequate description of the proposed AD, with clear rationale, scope, and its operational and statistical properties supported with tangible simulation evidence where necessary, may alleviate some cited concerns [2, 7, 11, 36]. This should include the appropriateness of the proposed AD to address the research question(s). Such description must also encompass the use and adequate reporting of appropriate, established statistical methods to control type 1 error and power and to obtain unbiased or bias-adjusted results (estimates of treatment effects, CIs and *P* values).

The use of retrospectively planned case studies aided with simulation work may help illustrate lost opportunities and provide assurance of the robustness of ADs in decision-making. This may also facilitate practical learning and highlight some pitfalls during the implementation of ADs. In addition, a review of undertaken ADs published in ‘high impact’ peer-reviewed journals and their publicity may help to improve acceptability of ADs in research to change clinical practice. Reassurance of the rigour in the science and conduct of ADs enhanced through transparent, adequate and accessible trial reports is paramount. Such accessible related trial materials include protocols (and amendments), simulation protocols and reports, open and closed IDMC minutes and interim results reports. Consumers of research findings should be able to make informed judgements about the quality of the AD in front of them. This can only be achieved through adequate transparent reporting that has been improved by the advent of CONSORT statements [57, 58]. Recent studies have suggested some modifications to the CONSORT guidance to accommodate ADs [12, 59], without, however, underpinning evidence regarding the state of reporting of ADs. We propose cross-disciplinary and cross-sector discussions to draw recommendations for a modified adaptive CONSORT statement.

We also recommend some form of standardised, consensus guidance toolkit tailored for the public sector (where interventions are so variable) similar to the guidance for the evaluation of complex interventions [60] that will address appropriate scope, benefits, statistical and practical considerations for successful implementation of ADs in confirmatory trials. This should be carefully crafted so as not to stifle design innovation. In addition, we propose the development of a troubleshooting toolkit tailored for trialists on important general and design-specific questions they should ask themselves when considering ADs at the planning stage. In our opinion, poor communication and dissemination deficiencies of AD methods to researchers are important perceived barriers, which require addressing. A generalised complexity message - ‘ADs are complex to design, implement and analyse’ - could act as a barrier, by deterring researchers from implementing even simple ADs because the level of complexity varies considerably across types of ADs. Hence, we believe there is scope for a toolkit addressing practical and statistical issues specific to particular forms of ADs, rather than the most common generic qualitative statements. We believe too much generalisation of AD-related issues is becoming a syndrome and a communication barrier scaring off some researchers.

We advocate for ADs to be considered alternative trial designs in the ‘toolbox’ of trialists and incorporated as part of MSc academic training curricula in Universities - targeting aspiring trialists and not just statisticians. It is also vital to raise awareness regarding the acceptable scope of Ads, when appropriate, among UK CTU trialists. We encourage collaboration among UK CTUs and industry organisations and the creation of a knowledge-sharing platform on AD-related issues to facilitate problem solving and learning from pacesetters. We support initiatives by UK public funders such as the NIHR and MRC on AD-related capacity building. We hope these efforts will enhance the capacity of experts with practical knowledge to help UK CTUs wishing to undertake efficient, but time consuming and complex ADs, when appropriate. These experts should also be available to undertake scientific peer-review of AD-related grant applications.

We support initiatives by public funders to communicate their receptiveness towards appropriate use of efficient designs such as ADs. We encourage more effort through outreach events to communicate this shift in position to trialists and launching of related funding research opportunities. In addition, we encourage knowledge and experience sharing among public funders to facilitate problem solving on AD-related issues, such as drawing up flexible contractual agreements suited to ADs. Refresher training of public funding panels and board members prior to their grants review meetings may improve their awareness on AD-related issues. More so, it is important for trialists to describe aspects of the proposed AD and related decision-making scenarios adequately in grant applications in order to help public funders to make informed decisions.

### Strengths and limitations of the study

The main strength of this study is that we have built upon previous research by incorporating interviews of key stakeholders and maximised sampling variation to capture perceptions and experiences that are more diverse to inform subsequent surveys to be reported elsewhere. This enhanced our robust exploration of barriers and related facilitators to the uptake of ADs. We also used an additional experienced qualitative researcher (JB) to validate consistency in annotation and coding of 7(26 %) of the transcripts, which is recommended as good practice in some qualitative research good practice checklists [34]. In addition, mapping of themes was discussed independently with experienced qualitative researchers (JB and Alicia O’Cathain).

One of the study limitations is the poor participation by health economists, which limited the exploration of AD-related issues among this stakeholder group. Non-participation could be due to a lack of basic understanding of ADs and their implications for health economic evaluation when trials are stopped early, and to some extent, health economists may feel on the periphery of clinical trial design. Thus, there is need for research to understand the implications of ADs on health economic evaluation. Finally, our study participants were predominantly from the UK public-funded setting - thus generalisation to other settings, particularly in relation to organisational barriers may be limited.

## Conclusions

There are still considerable, multifaceted individual and organisational barriers perceived to be hampering the appropriate use of ADs in publicly funded confirmatory trials Nevertheless, widespread interest and UK public funders’ perceived positive changes in attitudes and receptiveness towards ADs when appropriate are supportive, and a platform for the future use of ADs in the UK public sector. Our findings have been used to inform the design of follow-up surveys to be reported elsewhere, aiming to generalise these findings, rank barriers with respect to importance for prioritisation and to contrast themes on barriers between stakeholders based in private and public settings.

## Additional files

Additional file 1:
**‘Interview guide’;** ***Description of data*** **‘A guide to the interview process’.** (PDF 112 kb) Additional file 2:
**‘Supplementary interviews data supporting themes and shared case studies of adaptive designs’;** ***Description of data*** **‘Interviews quotations’.** (PDF 475 kb)

## Abbreviations

AD: Adaptive design
CRC: Clinical Research Collaboration
CRN: Clinical Research Networks
CTU: Clinical Trials Unit
DRF: Doctoral Research Fellowship
EMA: European Medicines Agency
FDA: Food and Drug Administration
GSD: group sequential design
HTA: Health Technology Assessment
IDMC: Independent Data Monitoring Committee
IQR: interquartile range
MAMS: multi-arm multi-stage
MRC: Medical Research Council
NIHR: National Institute for Health Research
NHS: National Health Service
NIH: National Institutes of Health
PhRMA: Pharmaceutical Research and Manufacturers of America
SSR: Sample size re-estimation
TSC: Trial Steering Committee
