# Supplementary material for: Missing steps in a staircase: a qualitative study of the perspectives of key stakeholders on the use of adaptive designs in confirmatory trials
Source: Trials. 2015 Sep 28;16:430. doi: 10.1186/s13063-015-0958-9 (PMC4587783; doi:10.1186/s13063-015-0958-9)
Supplement: Additional file 1: — ‘Interview guide’; Description of data ‘A guide to the interview process’. (PDF 112 kb) [file 13063_2015_958_MOESM1_ESM.pdf]

Interview guide

1.1 Introduction

Completion of the consent form and short baseline questionnaire were checked prior to all interviews.

- The interviewer begins by thanking interviewees for their willingness to take part and for sparing their time
- The interviewer briefly describes the overall aims and objectives of the research with its scope, what has been done so far and expected to be done on the future, what is expected from the interviewee during the interviews
- The interviewer reinforces that there are no right and wrong answer to the questions asked but it is about views and perceptions which are important.
- Participants were given a chance to ask the interviewer any study related questions before the start of the interview
- Interviewees were asked if they were ready for their interview and to begin audio recording the proceedings

1.2 Specific topics or subjects covered during the interview

1. Interviewee's primary roles and responsibilities in clinical trials research
2. Level of awareness, training and understanding of adaptive designs
3. Familiarity with opportunities or benefits associated with adaptive designs in confirmatory trials
4. Awareness and knowledge of adaptive designs which are applicable to be implemented in confirmatory trials
5. Personal views and attitudes regarding the use of adaptive designs and future prospects, especially in confirmatory trials
6. Perceptions regarding the use of adaptive designs by members of the research community

7. Views and attitudes towards the use of *ad hoc* versus planned adaptive designs in confirmatory trials
8. Perceptions about the accessibility of adaptive methodology and implementation resources by key stakeholders in clinical trials research
9. Perceptions about general challenges or obstacles hampering the use of adaptive designs where appropriate at the design, implementation and reporting stages (*prompting experience and solutions to barriers where possible*)
10. Perceptions about role specific challenges posed by the use of adaptive designs (*prompting experiences and solutions to barriers where possible*)
11. Perceptions about challenges specific to the public funded setting (*prompting experiences and solutions to barriers where possible)*
12. General concerns raised by the use of adaptive designs or specific adaptation
13. Experiences in the design, implementation and reporting of adaptive clinical trials (*prompting examples and lessons learned which could be shared with other clinical trialists where possible*)
14. Perceptions about credibility, validity and acceptability of the findings from an adaptive trial (*prompting specific adaptations*)
15. What should be done to improve the uptake of adaptive designs where appropriate in confirmatory setting

1.3 The end of the interview and final remarks

16. Is there anything you would like to add about adaptive designs or reiterate which you feel is important?
17. Interviewees given an option to receive a copy of their transcript for verification
18. Closing remarks by the interviewer thanking the interviewees for their contributions to the research and let them aware that summary of the findings will be made available to them in due course
