# Supplementary material for: Missing steps in a staircase: a qualitative study of the perspectives of key stakeholders on the use of adaptive designs in confirmatory trials
Source: Trials. 2015 Sep 28;16:430. doi: 10.1186/s13063-015-0958-9 (PMC4587783; doi:10.1186/s13063-015-0958-9)
Supplement: Additional file 2: — ‘Supplementary interviews data supporting themes and shared case studies of adaptive designs’; Description of data ‘Interviews quotations’. (PDF 475 kb) [file 13063_2015_958_MOESM2_ESM.pdf]

Supplementary interviews data supporting themes and shared case studies of ADs

Heading/Subheading	Additional supporting interview quotations
Perceived advantages of ADs	"I think it does potentially make it look more attractive project to funders because if you can say well if we did it to the end this trial would cost £3million but we think we can do it for half of that cost then that has to look a lot more attractive because you are showing better value for money for what they are going to put in. Also the time issues, the sooner you can get something out there and working in the NHS for a benefit then that has to be good.….Also, obviously from a patient point of view the sooner that if there is a drug or new intervention that is really effective then we want to get that into NHS practice, equally if it is dangerous or if there is anything that we shouldn't be using then we would want to get that out and into guidelines and into NHS practice as much as possible." *(QL01 CTU Deputy Director, Proposal Developer)*<br>"As a clinician researcher, so someone who has actually involved in delivering the research then the main advantage would be that it allows you to stop early and you don't have to recruit patients when there is clearly no benefit to the trial from doing that so it saves you work. I think the main advantages of adaptive designs accrue to funders because funders are not paying for essentially redundant data and ethically I think there is a benefit to patients because clearly we don't want to be recruiting patients to trials when there is no significant potential of that trial and additional data giving you any new information. I think when you are a patient participating in a trial hopefully the involvement in the trial does not involve any significant sacrifices but it still involves a certain amount of your time and effort to participate in it and the idea of taking part of the trial is that you are benefiting society by your participation, so if the trial has already given you the answer then you are going to the effort of participating in the trial without any benefits" *(QL04 Chief Investigator, Vice Chair –Public Funder)*<br>"Well, certainly, I guess it depends how well you know your drug up to that point. If it's a case of, 'well, we've done a dose-ranging study and we want to pick two doses from three, because there's a degree of uncertainty at the end of the dose-finding study but all doses appear to have an appropriate safety and efficacy profile to date, then perhaps the adaptive design where you drop a dose or possibly two halfway through the development - conditional on a huge amount of work being done up to that point – is perhaps a good use of resource … I think another aspect I think would

	be when you certainly have a limited patient pool for something like orphan disease implications where you know you're not going to be able to actually recruit sufficient patients for a full Phase 2/Phase 3 traditional development programme and we accept and understand that, as regulators and industry, you're offered appropriate incentives under orphan designation in the EU and various other – US and things. So I think there, there's undoubtedly a challenge – an opportunity to maximise the best use of patients, if it's selecting the right dose and then, or honing your patient population, or approaches where patients are in general limited. I mean, I have once actually seen a combined Phase 1/2/3 study, all in one go (laughs)!" *(QL16 Regulator)*<br>"My real focus is more efficient, better use of data within clinical trials, so for example, instead of just taking the data at the end of the trial and then having to reproduce or redo the trial to me the whole aspect is lets learn as we are going along and it has it benefits, not just in using all of the data but you can kill poor drugs early, you can advance good drugs early. So it is more a case of trying to understand the data and adjust your design according to the data that you are getting. One of the things that I always tend to tell people is that adaptive designs will make you address the objectives of interest so they will enable you to make the right decisions earlier rather than later, for example, the biggest opportunity is stopping poor drugs early, most of our drugs fail, 90% of the drugs that we start developing in phase1 never get to the full registration, we should be killing those drugs as early as possible and adaptive designs allow you to do that, whether it is in phase 2 or phase3 there is always that opportunity to stop early for futility." *(QL15 Statistician)*
Perceived therapeutic areas of opportunity to use ADs	"Well we have probably had applied it to most clinical areas, again group sequential design is fairly standard across almost entire portfolio – oncology, cardiovascular studies, respiratory studies and so on". *(QL11 Statistical Team Leader, Private Sector)*<br>"I don't think they're for every situation, I know sometimes with certain disease areas it's harder to employ them because they're very established (methodology) in these areas and the regulators aren't necessarily so keen on going down a novel route. As I said I think for like the rare diseases, it gives you a great opportunity to do more with the data and the information that you can collect and get more out of it to allow you to make your decisions with fewer subjects because you have fewer patients available to you" *(QL13 Statistician)*

	"I think for things like complex interventions where there is an awful lot going on and there is all sorts of factors effecting whether your intervention is going to be successful or not then it is probably harder to pin point what exactly is going on at an earlier stage and decide to drop part of that arm or drop that particular treatment so I think there is probably more work to try and work out whether or not it could be used for different designs rather than the more sort of traditional IMP drug trials think particularly with complex interventions you have got a lot of thing going on – ongoing process evaluation, there's different practice to do with who is delivering the intervention, in what setting. All of those kind of things are going to influence it and whether you can kind of separate that out at an early stage might be trickier than – if it is a drug, presuming you have got a particular dose – then you can say it's much easier to stick to that and to know that fidelity wise that the patients are doing what you think they are doing for the intervention." *(QL01 CTU Deputy Director, Proposal Developer)*<br>"So in emergency medicine it is potentially beneficial because we do measure short term outcomes, we do have to be careful not to overstate the benefit to emergency medicine because to a certain extent short term outcomes are interim outcomes, even something as important as survival, you might think that if you can show that there's a 5% increase in survival or there is not going to be any difference in survival, that would be a very valuable early outcome but of course survival is only meaningful if it translates into long term survival" *(QL04 Chief Investigator, Vice Chair – Public Funder)*<br>"So [name] introduced the concept of not just adaptive but Bayesian adaptive designs in the context of the ideal approach to how one might conduct RCTs of candidate interventions in the context of a rapidly evolving pandemic of influenza or influenza like illness with high case fatality." *(QL21 Chief Investigator)*<br>"I've also been involved in generally thinking through whether an adaptive design is more flexible for studying interventions in the critically ill during epidemics" *(QL22 Chief Investigator)*<br>"But I think with the more opportunities now to look across disease areas, so if we're looking in one particular area let's say, I don't know oncology, you could look at various disease types rather than just a single disease type. And …rare disease type areas where you've got very small populations worldwide who are experiencing the disease. I think there's a lot of opportunities there with adaptive designs" *(QL13 Statistician)*

Case study A	"Yes, it was a mild pandemic so I guess that gives you some context for what a bad pandemic would be like but in developed countries there was probably in the order of 200,000 to 300,000 patients admitted to intensive care with severe influenza and as best as we can work out about 30 – 40 were randomised in a controlled trial. The reason for that is that the lag time for setting up any sort of interventional trial is at least 6 – 12 months requiring funders to agree, ethics committees to approve, governance to be organised, study drug and study procedures, case report forms, randomisation project management and none of those things can be put together in time lines measured in weeks to a month or so. So part of the concept behind this Toronto meeting which has also been a now merged effort between the people who went to that Toronto meeting and a group called ISARIC (International Severe And Respiratory Illness Consortium) is the broad proposal that we should have "mothballed" randomised controlled trials that can be activated in a couple of weeks in the event of an arrival of a pandemic. So that means we would know the interventions that we are going to test, we would know the sites that are going to participate, they would ethics approval, case report form would be developed, the study drug and all other study procedures would be warehoused and able to be activated at short notice. But having gone to the point of feeling that that was a good idea, there was then a lot of really useful discussion about whether a classic frequentist design is optimal or whether some sort adaptive Bayesian design would be preferable. In a pandemic there is a rapidly rising number of patients with life threatening illness so a pandemic wave will typically last 6 – 10 weeks in any location but move around the world in different dynamics, so where an emergency is going to appear there is a time imperative to provide results to clinicians and policy makers. If you do a classic frequentist design you have got to make guesses about adequate sample size and your fixed to that design and then you are not able to utilise the information that is accrued to adapt the design as you go and so you could find that you have randomised 5000 patients and was being futile and proven to be futile up to 3000 or you could have randomised 3000 and found that you have got an effective intervention after 2000 and many patients who would have benefited from that knowledge and have been through the health care system without that knowledge being applied to their treatment. So there is this time imperative that doesn't so much exist where there is a stable incidence of disease, adaptation really comes to the fore as a design feature to generate the maximum amount of useful knowledge in a shortest possible period of time." *(QL21 Chief Investigator)*

Case study B	"So particularly the one particular trial that we're involved in a trial in tuberculosis using the multi –arm and multi - stage design. And it's a trial that's being conducted in South and East Africa. So there are lots of challenges … I suppose first of all the methodology itself was developed in Oncology. It was developed for a different disease area for a different set of end points (time-to-event). And so there's a little bit of work in adapt in the methodology to fit the particular disease area so in terms of what the end points were and some issues around how the analysis is done. So most of the methodologies around cancer where the end point is death - in tuberculosis the phase 2 end point is culture conversion. So there are a few minor tweaks like that. So the first challenge I guess was adapting methodology. We didn't have any problem in communicating (the design) to the other stakeholders (investigators) and the other investigators based in Africa. This was the right design and everyone seemed to appreciate that it seemed to be a sensible approach to use an adaptive design where we might be dropping arms that are not performing well. The five arm study was just four intervention arms with one driven analysis. I get challenges with data entry ensuring that the data was entered in a way that it could be collected on a central database in a rapid fashion and put it into analysis. So it could be cleaned and entered on an ongoing basis but at any one time you have real time access to the data. And so we've used tablet computers for data entry with a central database. It's clear that adaptive design is somewhat complicated to implement. And because of interim analysis there are obviously issues around controlling type one error but also controlling the power as well. You don't want to be stopping arms or indeed declaring arms; it's a bit efficacious too early. So there are concerns there. I think in our context the methodology was fairly well worked out so that helped. So I work in …..And so I have colleagues who are working on the design and methodology so it was use …….so the power of work on the methodology alongside running the trial. And so that helped to provide the necessary underpinning of methodology and to have confidence that it was working and that you know time and error was adequately controlled and that it made sense. I guess that's always a concern with adaptive design; it's a little bit more complicated than a traditional design. So in our study we had interim analysis so we looked at the data. We had an independent data monitoring committee who view the results. And based on the sort of pre-specified threshold they recommended stopping 2 arms. And so there were challenges in how to communicate that. We worked very carefully about how to communicate it. Explaining that it would that it was about lack of benefit rather than necessarily toxicity. So these arms are being dropped because there was insufficient evidence for benefit, not necessarily that they

	were bad arms or the patients were being put at risk or that they were necessarily inferior drugs. But just that in the context of the trail we're optimising resources and focusing on the other 2 arms rather than which arms are being dropped. There was a lot of discussion about how to present that. So that's definitely a challenge around you know how you present the results of or how you present the modifications during adaptive design" *(QL26 Statistician)*
Case study C	"Yes, so I have been involved in the design that's currently the [organisation] on a large multi-centre combined phase 2 /3 trial that is on the verge of having regulatory approval looking at a novel vasopressor agent. The patient relation remains static over time and there's a Bayesian priors enrolment but it involves a response adaptive randomisation in the beginning phase to try to narrow down the population … so we start the trial with a placebo arm and three dose arms and then there is a possibility of adding a fourth dose arm. The model that the design incorporates is essentially a model of dose response to try to gleam information from the overall behaviour of the drug across the different doses and that's used with a set of pre-trial assumptions about likely dose response so that in the absence of a dose response we will go with the lowest dose as long as there are no safety concern that is dose related and there appears to be efficacy it will tend to favour higher dose and in fact the fourth dose that could be added in is an even higher dose, it's like we haven't hit the ceiling. Then for the switch from phase 2 to phase 3 is variable depending on the behaviour in the opening part but the final half will still have a minimum number of patients into the final dose and the final placebo so that the total sample size is potentially variable because there could be a delay before we choose the dose if there isn't much of a difference in the dose so the dose selection study is variable in size but the second half will have minimum number of patients. It's going through European regulatory approval right now but hasn't been received, so it's in the planning phase.<br>I've also been involved in generally thinking through whether an adaptive design is more flexible for studying interventions in the critically ill during epidemics. A trial could be up and running, targeting a broad population but could then be more flexible to adjust potentially by bringing in new study arms in the middle of an epidemic as opposed to trying to launch the trial at the time the epidemic starts so seeing whether adaptive design is more suitable for just in time research if that makes sense.<br>Probably the biggest regulatory hurdle has been a discussion around – there's a couple – one is around controlling alpha (type 1) error and the concern that it's not that easy to generate empirically but rather you have to

	run simulations and there's a discussion about whether the simulations truly model across the entire space of the trial. So whether it is giving a robust understanding of the alpha error, that has been a concern. Another concern is that if you are recruiting placebo patients all the way through but you disproportionately recruit intervention patients into the final arm more heavily towards the end of the trial then there is the risk that if there is some sort of time bias across the trial then, if for example people get better at caring for these patients later or if there's changes in case mix then the comparison may not be taking the full advantage of trying to use true randomisation to hope that you would have a balanced distribution of both known and unknown covariance at baseline. Then a third hurdle is the overall combined trial most definitely speaks to whether drug on average is superior to placebo but it's less clear that it really gives a clear statement about whether the particular dose is better. You know, you could argue that the statement about dose can only really be made about the later patients enrolled in that arm and so there is a question about what would be the label that one could right from the results of the trial. I guess another issue is using a Bayesian framework, it does not necessarily return a classic p-value, which does not necessarily concern the regulatory authorities but it might be hard for clinicians to understand what that means. So those are some of the hurdles which I think are relatively generic but those are some of the issues we have been discussing at length with the regulatory authorities" *(QL22 Chief Investigator)*
Cross-disciplinary lack of awareness and understanding<br>- Perceived underutilisation of ADs	"Probably the main thing I am aware of is that I am acutely aware of the fact that we don't use them very much and I would really like to think about how we could be using them more so I would not probably put myself forward as someone whose got experience of designing adaptive designs but I am aware that we are probably not being as efficient as we could be in a number of our studies and it would be useful to think about where it is, which is why I was interested in your project." *(QL09 CTU Director, Statistician, Funding Panel Member)*<br>"In the context of the last pandemic there was a massive missed research opportunity, there were about 200,000 deaths worldwide from influenza." *(QL21 Chief Investigator)*
Regulatory receptiveness and improving awareness and experiences<br>▪ Regulatory caveats	"I think what you want to get out of it, there are 2 broad aspects, you want the type 1 error for the trial to be good so you don't want to be making false positive decisions any more often than you would be doing otherwise if there hadn't been an adaptation, I think this is covered very well in that you see a lot of papers and when people come in they have put a lot of thought into the type 1 error controls so I think that is generally thought of greatly and covered a lot

	and so you can be pretty sure that p-value at the end is going to represent the data accurately, people are very concerned about that. What seems to receive less attention is getting an unbiased estimate and getting a confidence interval around that estimate and that is important as well for decision making and depending on adaptive designs – there comes a wide variety of things – there can be some adaptive designs where it's difficult to get that from it and I would say that less thought is generally giving to that and people sometimes seem to think that when they have controlled the type 1 error then that's job done. The size of the estimate and the confidences around it are very important and can be difficult in the face of some adaptions*" (QL19 Regulator)*
Confusion over what is meant by an AD<br><br>▪ Whether modification to the intervention is within scope of phase 3	"One of the things which I'm not sure is an AD, but I think is an issue, is the pace of change interventions and I'm not really sure this is what you'd call an AD,…I suppose the more I'm talking about this, the more I'm realising I'm not really sure what an adaptive design is to be honest because, … an internal pilot you don't expect the intervention to change, so your intervention is pretty much static and you're mainly focusing on recruitment and maybe your sample size. So that's pretty much it. I would say that AD, you're not really focusing on recruitment, I wouldn't have thought that was an issue with an AD" *(QL01 CTU director)*<br>"The first part of the guidance is to describe clarification to the clinical trials community of what an AD is. You can read 12 different versions of it and I think the first thing is to clarify what we mean by an AD because in reality – because you know …in reality almost all of our trials funded nowadays have an adapted element or an element that you could call adaptive so for example – over the last 15 years I would say we are doing far more pragmatic clinical trials that now involve a preliminary internal pilot randomised control trial, which will have a feasibility element – can they recruit enough people, can they retain enough people and it may actually also include an element of perhaps assessing the control event rate to reassess the sample size so in the broader definition of AD I would say it is an AD." *(QL25 Chair –Public Funder)*
Cross-disciplinary lack of awareness and understanding	"It has got to be done on an individual trial by trial basis trying to understand what sort of adaptation in the current state of knowledge that stops you from designing the final phase 3 fixed designs now and learning as you go along" *(QL07 Statistician)*<br>"Certainly you become aware that industry are very keen on adaptive designs from the efficiency point of view. But of course I think that ups some people's anxieties, and then...that people who are very anti it, and that's usually just

	that complete lack of understanding of the notion of using trial data as trials go along, looking at the data as it's happening." *(QL08 CTU Director)*
Against conducting too many adaptations in a single trial<br><br>▪ Limited scope in confirmatory setting	"In phase 3 if you throw loads of different adaptations at it then you can get yourself into a right mess with stuff that you can't really interpret at the end and it can be difficult to run. So you need to think about what you are trying to achieve and if you have got some uncertainties by all means do something like this but make sure you really need to do it and not just for the sake of it." *(QL19 Statistician)*<br>"Well phase 3 is less likely to use them really, in a strict sense it is just merely there to confirm a result and meet the regulatory guidelines. I know the FDA are pretty much - well there's nothing ever set in stone, the FDA never set anything in stone from a legal perspective but the consensus is generally that they don't like doing adaptive designs. I suppose by the time you get to a phase 3 they shouldn't really be in a position to need an adaptive study, you should have all of the information that is needed to do the most efficient study that is of a fixed design I suppose." *(QL7 Statistician)*
Cross-disciplinary conservatism<br><br>▪ Regulatory buy in reluctance	"I think the question about regulatory buy in probably is more important and the concerns that a trial may not get accepted because maybe you have done something in the design that introduces bias or some other concern." *(QL12 Clinical Research leader, Trial methodologist)*<br>"…We usually try and discourage companies from using adaptive designs at Phase 3, because at Phase 3, we're hoping that will be the confirmation that the drug works and they should have developed all the theories of how the drug works for the best dose in Phase 1, Phase 2. By the time you get to Phase 3, if you're still not sure about things it seems to me that you shouldn't be in Phase 3. And then it gets tricky – depending on what adaption you do, if it's just a simple interim analysis to stop early, that's not so bad but if you're still trying to decide what's the optimum dose, then it's a bit tricky ... if we get a study and it's had adaptations but it looks robust – and what we would look at, if there have been interim analyses and there have been changes along the way, we would want to see that there haven't been huge changes in the estimate or in the population involved that would indicate that some of the results got leaked or the investigators knew this interim analysis was happening and they knew what the consequences would be and they can infer from the study continuing whether it's going well or not so well depending on what the interim analysis

	was supposed to do, so if then we end up with a treatment effect that the difference between, say, placebo and treatment didn't look that impressive up until interim, not because it was underpowered but the actual, you know the estimate rather than the confidence intervals, and then suddenly it looks huge at the end and we can see that, I don't know, suddenly, they started recruiting better patients or - that's where we start getting suspicious and then what do we do?" *(QL18 Regulator)*<br>"I don't have as many concerns as the regulators do, I think the regulators don't like to reduce the sample size. I think where that can be argued is in a situation where it is a rare disease, obviously there is a limited number of patients you can use and therefore I think a reduction in sample size might be used." *(QL15 Statistician)*
Cross-disciplinary conservatism<br>▪ Type of the proposed adaptation and how perceived and established the methodology is	"I mentioned GSDs, they are well accepted, they have been used in phase 3 designs, they have been around for many years now, there is lots of published material for GSDs ...The other ones are any stops to futility, which should be acceptable in phase 3, if a drug doesn't work let's stop it. Blinded SSRs are acceptable in phase 3 according to the FDA, what I mean by that is a sample size increase not decrease based on some overall estimate of variability or some overall number of events, so not broken down by treatment group, if you break it down by treatment group that is when you start introducing bias, so it is always a blinded situation." *(QL15 Statistician)*<br>"I'm much more comfortable now with the idea of maybe setting out, even on a phase 3 trial, with 4 or 5 potential interventions and dropping the ones that look least promising" *(QL14 Statistician)*
Cross-disciplinary conservatism<br>▪ Concern about impact on important secondary objectives	"I suppose the limitation of adaptive design is that it may focus on your primary end point and so perhaps lead to a study stopping on the basis of a primary end point whereas you may also as a clinician be very interested in the secondary endpoints, so a typical cardiovascular study will look at the combined end point of cardiovascular death, stroke and myocardial infarction so obviously cardiovascular and death is more important than non-fatal myocardial infarction and if you stop early a study based on a benefit in terms of reducing the primary end point driven primarily by non-fatal myocardial infarction then it will limit your ability to detect a difference in the secondary endpoint of cardiovascular death which most clinicians would see as the most important end point. So I think that is one of the limitations in that it needs to be explored and acknowledged in a trial design"*(QL24 Chief Investigator)*<br>"… With a slight caveat normally the thing that would drive the health economic results is the very wide confidence intervals around the efficacy (estimate). So if you didn't follow it up long enough and the one that I will send you -

	there it depended (on) the log logistic I think it was, or it might just be a logistic and a Weibull distribution but they were massively different but if all you have done is stopped really early and you have got no feel for what is going to happen to the tail you could cause uncertainties so that is the one side but typically if it has been followed up for a long enough period that the clinicians are happy they may also have to see that there is some form of proportional hazards, or you know that they can be sure the effect is maintained. So I am assuming that the clinical experts have done a good job when they say they know for certain so the only thing would be if data collection stopped at something very short, 6 months *(QL29 Health Economist)*
Cross-conservatism<br>▪ Concerns about inferential seamless 2/3 AD	"The one that from our perspective always caused the most concern, without a shadow of a doubt, was a Phase 2, Phase 3 seamless process where the data from one half of the trial was then merged with the second half of the trial and used effectively as confirmatory evidence in and of itself. And I think there was perhaps nervousness that this would not be necessarily be appropriate or how we would handle the data from the two halves of the trial… If you have got an AD which is seamless in execution but not seamless in analysis so it's got the 2 phases … and if you selected the dosing in one phase and then done an independent analysis for the doses that actually went forward then you are confirming a hypothesis and it's irrelevant that it was done in a seamless and adaptive fashion, it gets a bit more concerning when you are basing it on combined data so the data that you use to generate your hypothesis is also somehow there in the confirmatory test…" *(QL16, Regulator)*
Cross-disciplinary conservatism<br>▪ Concern about acceptability of adaptive designs in decision making to change practice<br>▪ Concern about robustness of adaptive designs in decision making	"I think there is a potential concern in the clinical community that they may struggle with the acceptability and I think a lot of this is to do with what we are used to and accepted precedent that if we see a trial that has been stopped early we tend to assume that something must have gone wrong or that it must have failed in some way and that we should therefore discount the findings, whereas clearly that is not what you want from adaptive design. If you stop early for whatever reason, you want to stop early knowing that you have answered the question and I think to ensure that you have answered the question you need to be able to convince the readership, convince journals that this is a definitive answer even though it has stopped early. I think there is a fairly crude assumption often made by journal editors, reviews and clinicians that if it is stopped early it cannot possibly have provided us with a definitive answer" *(QL04 Chief Investigator, Vice Chair – Public Funder)*

	"‥ the first is that the purpose of clinical trials is to provide evidence to clinicians and policy makers for changing practice and policy so ultimately the design of trials has to be crucially dependant on whether or not the consumers of that information will change their behaviour depending on the results. So adaptive trials are relatively new and clinicians and policy makers are often conservative and if there was clear evidence that they would not change their practice irrespective of the strength of the result that came from an adaptive design then I think that leaves a substantial question mark about the appropriateness of using adaptive designs" *(QL21 Clinical Investigator)*<br>"I don't know if I know of any (ADs) that I would discourage, I suppose as long as it is done appropriately so you don't want people making decisions based on bad evidence or making a decision… I suppose the problem with adaptive designs is that if you make a decision early in a trial and it is not based on robust evidence then you have got the danger of stopping the trial early which could have later on shown to be effective or vice-versa, you might be saying that you think something is effective when in fact it is not, so I guess it is just ensuring that it is designed and appropriately analysed so that you are not stopping for the wrong reasons." *(QL01 CTU Deputy Director, Proposal Developer)"*
Concern about population drift	"I think if you ran a trial and at the end of the trial the patient number was relatively large and the patient population looked like the population of interest and there wasn't a concern that there was big change in the patient population over the duration of the trial and observed event rates of the primary outcome were impressively different, if it was a very large effect that looked biologically plausible then I think clinicians will not worry about the methods. If on the other hand the result is more borderline then the acceptance may be less clear" *(QL22 Chief Investigator)*
Cross-disciplinary conservatism<br>▪ Unfamiliarity and lack of understanding	"I guess another big issue is unfamiliarity with them and DMECs and TSCs will have got used to ways of working and rules apply in informal ways and the general fairly sound idea that we should not be looking at data unless we have to and so actually accepting that it might be appropriate to have looks at data is often a challenge to steering committees and DMECs" *(QL04 Chief investigator)*<br>"I think that (AD) ups some people's anxieties, and then...that people who are very anti it, and that's usually just that complete lack of understanding of the notion of using trial data as trials go along, looking at the data as it's happening" *(QL8 CTU Director)*

Lack of knowledge and experience	"…Ethics committees are not so aware or educated and are very unlikely to, even if there is a clear case of a highly inefficient design going on that could be improved by an AD, the Ethics Committee are never going to stop it going through on such grounds, even though in principal they should, they should say that this is unethical because you will be prolonging a potentially less effective treatment by sticking to your rigid design." *(QL7 Statistician)*<br>"There has been a little bit starting to come through (training) but I think the majority of it is that it has been based at statisticians I think so far and so more accessible training for those of us involved in designs and understanding the design issues and practicalities as well would be really useful" *(QL01 CTU Deputy Director, Proposal Developer)"*
Cross-disciplinary conservatism by funders<br><br>- Unfamiliarity<br>- Uncertainty	"I think there is still a little bit of conservatism within the funding board with people who are less familiar with adaptive designs or feel maybe a little bit uncomfortable about the risks associated with going down an unknown pathway because you have to remember funders are always allocating money in competition with other people and if you get an application for an adaptive design saying "we will do an interim analysis after 12-18 months and our sample size may be anything from 400-1200 and the cost of the study is already £1.6million based on 600 people, then it is very difficult for us to go into a sort of bottomless pit / open ended arrangement with the trials community so that they keep coming back at us saying "oh we want to add in another arm, actually it's 1200 patients and by the way it's now going to cost £4.5million" *(QL04 Chief Investigator, Vice Chair – Public Funder)*
Degree of statistical and practical complexities	"So, to write the proposal it's really complicated because you've got to set all these different scenarios; if this happens then it's going to need this many centres and that much resources, but if that happens then this and that. So instead of having one design you've got about three, and you've kind of got to put the worst case scenario, so you've got this massive great number that might put the funders off … and at the moment, there's no scope within the application form to have a range (of costs)" *(QL2 CTU Director)*<br>"From a practical point of view when you are designing adaptive trials there is more work involved for the application in planning the trial and working out the timelines … you have to do it for a number of different scenarios. So the work involved in that is more from the trialist and statistician's point of view, the statistician has to do various modelling and look at different scenarios and we have to do all of the different planning and you are usually on a fairly tight deadline for applications because of the way that NIHR funding works so if you only have 6 weeks to

	work with the team, trying to fit in time to do lots of different scenarios can be quite tricky and can make it more difficult." *(QL01 CTU Deputy Director)*
Confidentiality and implications of ADs on IDMC duties and responsibilities	"It creates problems around ensuring that any looking at the data is done appropriately and there are the simple practical problems of making sure the trial team collecting the data blinding it in an appropriate way to send to the DMEC, the DMEC looking at it without revealing anything, the DMEC making sure that their discussions are held in confidence and are not revealed to the trial team and with the best will in the world there is always the risk of something going wrong at some point. I have been in the open part of a DMEC when the statistician has suddenly grabbed all the papers as he suddenly realised he had given the wrong papers to the trial team and were about to see unblinded data" *(QL04 Chief Investigator, Vice Chair –Public Funder)*
Robust data management infrastructure	"It's a lovely notion but you actually work with the NHS – and you know, I love the NHS, I don't want to be seen in any way as dismissive of the NHS – we actually have to build our web-based data entry systems at a very low technical level so that they can talk to the NHS and the NHS can use them. So there is without doubt what I would consider to be a technological challenge because I would worry about either. The data that should be coming in to inform the design as you go along not coming in quickly enough or not coming in correctly - it would worry me. If you're using as I understand the adaptive designs that I've been most drawn to, and interested about, you're using that data real time – almost - sort of type thing, to inform the design, and so how would you then be sure that the equivalent of that data management, data lock, data validation – you know what I mean" *(QL08 CTU Director)*
Concerns around trial credibility, integrity and validity	"I have come across cases where it was a case of a drugs firm saying "we are going to choose the doses as we go along without giving any further indication about how this process was going to be applied" they just had to come back with more details to say that they had a plan, it wasn't just stick your finger in the air" *(QL07 Statistician)*<br>"I have problems here (re reporting). Sometimes companies tell me they have done an interim analysis and I don't see – that's all they tell me and I want to see the report, there must have been a report at the interim analysis, to someone, to me or whoever it was, and often that gets just blurred somewhere and you have to search for it, and definitely maybe would increase, if it was written more clearly what happens at the interim analysis and the decisions that are made" *(QL18 Regulator)*

	"You need to have the whole history of the protocol and the changes and things and whether the adaptations were actually pre-planned from day one and all the back catalogue of DMEC reports and the open and closed things ultimately. Obviously no one is going to want to publish this in a Lancet paper, you want to see the headlines but there's lots of additional supporting material that should be available online or part of the open access for trials being completely transparent about what happened and what information was made available when." *(QL07 Statistician)*
**Public sector perspective**<br><br>• Worry about impact of trial early stopping on staff research contracts<br><br>• Complexities in marketing complex adaptive designs to key stakeholders<br><br>• Lack of capacity and expertise within UK CTUs<br><br>• Limitation of the grant application process	"There is a couple of different reasons, one is just the lack of expertise within the unit, so it is easier when you are very very busy to put forward a design you know rather than one you don't. It's also easier because if you put forward a design that does not look the same to clinicians who expect straight forward designs you have to be very very confident in that design to be able to convince them to some extent. Then the other aspect I think is once we are based in the University as a CTU we're completely run on external funding and actually my sense of adaptive designs is that you can be more efficient in your study but you have to do an awful lot more design work and at the moment none of that is funded so there are a few things that we are trying to develop at the moment that I am hoping will be adaptive designs but most clinicians want you to be working up a funding application and submitting it within 4-6 months when actually you need to put quite a lot of stats time on. I would need to free someone up quite a lot to do quite a lot of simulation work, certainly when it's the first time they've done those sorts of things and to be honest in terms of business case it is quite hard to argue for that as things currently stand" *(QL09 CTU Director, Statistician)*<br><br>"The first stage is the design of adaptive design so I don't think we've got enough… obviously there's [name], but as you know there's only a few of us that are supporting investigators to design studies and I don't know how much knowledge [name] has about adaptive design, but I don't have very much. So for one thing we need to have more knowledge so we're actually designing adaptive designs and knowing whether they are appropriate or not appropriate." *(QL02 CTU Director)*<br><br>"I think the challenge is to explain that at outline in a very concise way and part of that is its unfamiliarity so it is trying to break through a barrier, so if you put in a study design for a straight forward 2 or even 3 armed randomised controlled design, or even a factorial to be honest, most people on the panel know what one is so you don't have to

	use your words explaining that, you can just say it is a factorial design and everyone has an inherent common standard of knowledge of what that means so you don't have to use your small number of characters going into what it is, but I think some of the more adaptive designs people are less aware of what the words mean therefore it is very very hard. I find it quite fascinating how the NIHR have gone into an outline application form which is something like 45 pages but actually there is still so little space to write about method." *(QL09 CTU Director, Statistician)*
Facilitators to some barriers	"I find a lot of it seems to be driven by statisticians and some of the quite theoretical statisticians, some of them are very accessible but I think putting less of a stats hat on and more of Director of CTU hat on I think the logistics of running them would be…there has just been a paper published in Trials hasn't there around some of the practical logistical issues with running them so those statistical issues I think I have been talking with the stats group about the design aspects but we haven't really talked through the logistical aspects of those adaptations. Probably having a better understanding of how the areas that currently do it manage things like – how do you get 11 or 100 centres to change doing something that quickly, do you get a nightmare with R&D which is obviously always the issue, do you get a nightmare with pharmacies, what are the ways to actually, it should be more efficient but actually if you just leave yourself open to lots of people not following what they should be doing." *(QL09 CTU Director, Statistician)*<br><br>"In terms of funders I think it's important that we as proposal submissions, as investigators, we know what they want, because obviously you don't want to submit anything that they're not going to want to see. So it would be if they made clarity about pilot trials and what they expect to see in a pilot trial, the scope of it and even the funding levels, it will be good to see that from the funders. So up front about what they want to see adaptive designs, the sort of level and scope that they want to see, the limitations and the range, timescales, at what points they are wanting to see those decisions being made. So I think that would be helpful from them. I also like to see from them, sort of, reassurance about the process is going to work because, you know, to take the risk out of it for us it's important to know how long those decisions are going to be made and how long it's going to take and will the trial continue while they're making those decisions or will it just stop? That's sort of thing so we can plan for that because, you know, if it's going to continue you need to know quickly, you can't have a great big gap otherwise you can't, you lose your recruitment. So

	that's I suppose about the process. Something on the scope, something on the process. From other investigators with experience, such as the health workshop, the health methodology groups. A bit of sharing of experience, so pulling together those other people with experience of running adaptive designs so that we can learn from them and share some of the experience that has been had so far. In terms of methodology and design, but also practical issues of implementation and management within, you know, units and in teams, budgeting and all that sort of thing. So I think that would be helpful.<br><br>We've had ones (guidance documents) for complex interventions and we now got reporting for pilot trials, I think that's just under development, then, yeah I think we are going to need clear guidance on reporting for adaptive designs… So, we need sort of training, workshop on the statistical issues around analysis and interpretation and people who are going to be making the decisions on DMECs and things, they are going to need to understand these issues as well. I mean, maybe Statisticians just understand these issues, but you know, it's increasingly difficult to get people on DMECs so the people going forward are probably more junior, so they need to be familiar with these concepts and how to make these decisions." *(QL09 CTU Director)*<br><br>"I think the most useful thing or the best way to overcoming a barrier are to have examples of adaptive designs that have worked. I mean if a board like HTA were to commission a trial with an adaptive design that was then able to stop early and show a definitive result that is accepted by the clinical community then that is very powerful because then you can say to everyone, we really should use more adaptive designs in future because it could be like this trial that was published in the New England Journal and then you can say to people it then becomes something that people become more accepting of, clinical communities will say "clearly if this adaptive design trial got published in The Lancet or New England Journal then they are acceptable and we ought to not be worried about them". I think until you have that, people are always going to be suspicious. That is a question worth looking at. How many adaptively designed trials have there been published in the big medical journals? Are there any examples you can cite? It is always very powerful to be able to say, there is nothing wrong with this design, look at this great example that was published in The Lancet and then people say "okay". Whereas if there aren't any then the critics are always going to

	say "how many adaptive designs do you see in The Lancet or New England Journal?- None. So why should I be doing an adaptive design?" I think there was the same problem with pragmatic trials. The pragmatic trials struggled for acceptability, there used to be a perception that if you did a trial properly it had to be a tight experimental control you know very rigorous inclusion/exclusion criteria and very rigorous controlled protocols you know, not including anyone over the ages of 70 or anyone who have comorbidities and then that changed. I think it changed because we are able to point to big trials like the CRASH trial and say "look at these big pragmatic simple designs that have answered really important questions and got published in The Lancet" and such like. Then people say "yes we accept pragmatic trials are good and we ought to be doing them" and then you get acceptance. I think until you break through that you are going to struggle." *(QL04 Chief Investigator, Vice Chair –Public Funder)*<br><br>"I really think there must be a basic and fundamental change and I'm not sure about how to achieve this change, but I would like to have something like a more formalised discussion throughout a trial, and to have funding committees that really are able to judge adaptive design and the quality of applications of adaptive designs, so that they can release funding and say 'yes, we know what you're doing'. I mean, the problem is, if we would say we are doing an adaptive trial, we will evaluate for example sample size, it will be increased, but if then the funding committee would say, 'well, give us the data', then there would be additional delay so this should not lead to a delay, but we need a process that still makes the funders aware of what needs to be done in trials, so in terms of, yep, adaptations of funding" *(QL05 Statistical Team Leader)*<br><br>"That would be very good yeah. We talked a bit about planned analysis and what should be in and everything so consort type guidelines to know what you need to do ahead of time, so what should be in your protocol, what should be pre-planned to make it a transparent process so that even though you are doing a number of analyses it is still not going to affect people trusting your trial results at the end or thinking that you have found something that's just by chance – so guidelines on that and also on the reporting would be really good and yeah the groups, the consorts have done really well with that in spirit and everything so that kind of idea around adaptive designs would be great" *(QL01 Deputy CTU Director, Proposal Developer)*

	"So I'm general in favour, however convincing the community of that takes some work so a big threat for adaptive designs is just that it's a cutesy word which means different things to different people, there's misinformation about it and there are some existing biases in the community and so there really needs to be a lot of education. Education coupled with, there needs to be more concrete examples or anecdotes that clearly show ways in which an adaptive trial has outperformed a traditional trial. So traditional trials compared to observational studies there's a NICE literature showing the limitations of observational studies, even though I love doing observational studies there's lots of anecdotes of the way in which an observational study [..inaudible..] indication bias, selection bias etc providing misleading information and an RCT with a more powerful lever for causal inference provided superior quality information. The same thing needs to be true for adaptive trials, there needs to be instances where you could say "the following clinical question was tackled both by traditional RCTs and by some adaptive RCTs and look – the traditional RCTs because of the rigidity gave misleading information and the adaptive trials gave useful information" I don't think enough of those actual cases have been made and so you can teach about the theoretical advantages but until there is a real body of practical advantages it still remains a slightly harder trial." *(QL22 Chief Investigator)*
Key limitation<br>• Unfamiliarity with ADs and their impact on health economics evaluation	"… They (health economists) do not know enough about it (adaptive designs and their impact) and I would put myself in there as well. So I know bits about it but nowhere near as much to call myself even fairly good at it. Health economics is this really tight little world where quite a lot of people know all the rest of them, we used to do almost. It's not as bad as this, but we do quite standard or repetitive sets of models. And the adaptive ones have not come in even with the trial that I have reviewed, … and I have been on the NICE committee now for 4 or 5 years" *(QL29 Health Economist)*

Caveat emptor: Please note that participant's preferences and views on certain aspects may not represent facts.
